# Engineering strategies to address immune and delivery barriers in pancreatic ductal adenocarcinoma: a barrier-matched translational framework

**DOI:** 10.3389/fimmu.2026.1900936

**Published:** 2026-09-14

**Authors:** Yang Li, Yu Li, Jia Fan, Zhi-qiang San, Jun-feng Ye

**Affiliations:** 1 General Surgery Center, First Hospital of Jilin University, Changchun, Jilin, China; 2 School of Nursing, Jilin University, Changchun, China

**Keywords:** engineered cellular immunotherapy, immune exclusion, oncolytic virotherapy, pancreatic ductal adenocarcinoma, tumor microenvironment

## Abstract

Pancreatic ductal adenocarcinoma (PDAC) remains one of the most lethal malignancies, largely owing to profound therapeutic resistance driven by tumor heterogeneity, a highly immunosuppressive tumor microenvironment (TME), dense desmoplastic stroma, immune exclusion, and impaired antitumor immune surveillance. Although conventional chemotherapy provides limited clinical benefit, immune-based therapies have shown modest efficacy in unselected PDAC, highlighting the need for strategies that overcome the biological barriers underlying immune resistance. Recent advances in precision medicine and bioengineering have generated a diverse range of therapeutic platforms aimed at remodeling the PDAC ecosystem. This review evaluates oncolytic virotherapy, gene-editing technologies, engineered immune-cell therapies, nanotechnology-enabled delivery systems, and artificial intelligence (AI)-assisted precision oncology according to the PDAC barriers they are intended to address and the maturity of the supporting evidence. Oncolytic viruses may enhance tumor immunogenicity and reshape suppressive immune niches, whereas gene editing and engineered cellular therapies provide opportunities to target oncogenic vulnerabilities, improve immune-cell function, and overcome antigenic and stromal constraints. Nanotechnology-based platforms can modify tissue access, payload exposure, and local immune modulation in selected models, whereas AI approaches support molecular and spatial stratification and generate treatment-prioritization hypotheses. Most supporting evidence remains preclinical or early phase, and no platform class has established broad comparative clinical benefit in unselected PDAC. Translation remains constrained by intratumoral heterogeneity, delivery limitations, safety, manufacturing complexity, and insufficient predictive biomarkers. Translation will depend on biomarker-defined enrollment and on linking administered dose to tumor exposure, target engagement, biological activity, safety, and the added benefit of the investigational component. We therefore present a barrier-matched development framework rather than a validated treatment-assignment algorithm.

## 1 Introduction

Pancreatic ductal adenocarcinoma (PDAC), accounting for more than 90% of pancreatic malignancies, remains one of the most lethal solid tumors worldwide (1). Despite advances in surgery and systemic therapy, long-term survival remains poor because most patients present with unresectable disease, recurrence is common, and treatment benefit is limited by toxicity and acquired resistance (1, 2). Modified FOLFIRINOX and gemcitabine-based regimens improve outcomes in selected patients but do not overcome the biological determinants of treatment failure. These limitations make the mechanisms that sustain resistance a central problem in PDAC management. Increasing evidence indicates that therapeutic resistance in PDAC cannot be explained solely by tumor-cell-intrinsic abnormalities but instead arises from a complex and dynamic tumor ecosystem. PDAC is characterized by extensive genomic alterations, including frequent *KRAS* activation and inactivation of *TP53*, *CDKN2A*, and *SMAD4*, together with substantial interpatient and intratumoral heterogeneity. These molecular abnormalities are accompanied by diverse cellular states, epithelial-mesenchymal plasticity, and adaptive changes within the surrounding tumor microenvironment (TME). The PDAC TME is distinguished by dense desmoplastic stroma, extracellular matrix accumulation, heterogeneous cancer-associated fibroblast (CAF) states, dysfunctional antigen presentation, immunosuppressive myeloid populations, and limited infiltration of effective antitumor lymphocytes. Collectively, these features establish a “cold” immune landscape characterized not only by immune suppression but also by physical and functional immune exclusion, thereby preventing effective immune recognition and therapeutic response. The failure of conventional immunotherapies in unselected PDAC further highlights the importance of addressing these biological barriers. Immune checkpoint inhibitors targeting PD-1/PD-L1 or CTLA-4 have demonstrated remarkable success in several malignancies but have shown limited activity in the majority of PDAC patients. This resistance is associated with multiple interconnected mechanisms, including impaired antigen presentation, inadequate T-cell priming, stromal restriction of immune-cell trafficking, and tumor-driven remodeling of myeloid and fibroblast compartments. Nevertheless, biomarker-defined subsets, such as patients with mismatch repair deficiency or microsatellite instability-high tumors, indicate that PDAC is not uniformly immune resistant and that appropriately selected patients may benefit from immune-based interventions. These observations emphasize the necessity of developing precision strategies that can remodel the tumor ecosystem, enhance immune accessibility, and match therapeutic approaches to specific biological vulnerabilities. Recent advances in bioengineering, molecular medicine, and computational technologies have created new opportunities to overcome these barriers. Rather than directly targeting malignant cells alone, emerging therapeutic platforms aim to reprogram the PDAC ecosystem through complementary mechanisms. Oncolytic viruses may increase tumor immunogenicity, induce immunogenic cell death, and reshape suppressive immune niches. Gene-editing technologies provide opportunities to manipulate oncogenic pathways, regulate immune responses, and support target validation and the development of therapeutic products. Engineered immune-cell therapies, including chimeric antigen receptor T-cell (CAR-T), T-cell receptor-engineered T-cell (TCR-T), and related approaches, seek to overcome antigen heterogeneity, improve tumor trafficking, and enhance immune-cell persistence within hostile microenvironments. Nanotechnology-based delivery systems offer strategies to improve spatial drug distribution, transport immune modulators, and overcome stromal barriers. Meanwhile, artificial intelligence (AI)-assisted precision oncology enables integration of molecular, pathological, and spatial information for improved patient stratification, biomarker discovery, and treatment prioritization.

Most evidence for these platforms remains preclinical or early phase, and its interpretation is limited by model context, delivery, safety, manufacturing, and biomarker uncertainty. This review therefore evaluates oncolytic virotherapy, gene editing, engineered immune-cell therapies, nanotechnology, and AI within a barrier-matched translational framework. Rather than treating these modalities as independent categories, we ask which PDAC barrier each platform is intended to modify, what model can test that function, and what evidence is required to link administration to tumor exposure, biological activity, and clinical benefit. The review proceeds from PDAC biology and the clinical immunotherapy landscape to platform-specific evidence and a cross-platform development framework (**Figure 1**). Unlike reviews organized primarily by viral backbone, cellular target, nanomaterial class, or individual immunotherapy modality, this review compares these platforms according to the PDAC barrier they are intended to modify and the evidence required to demonstrate that function.

**Figure 1:**
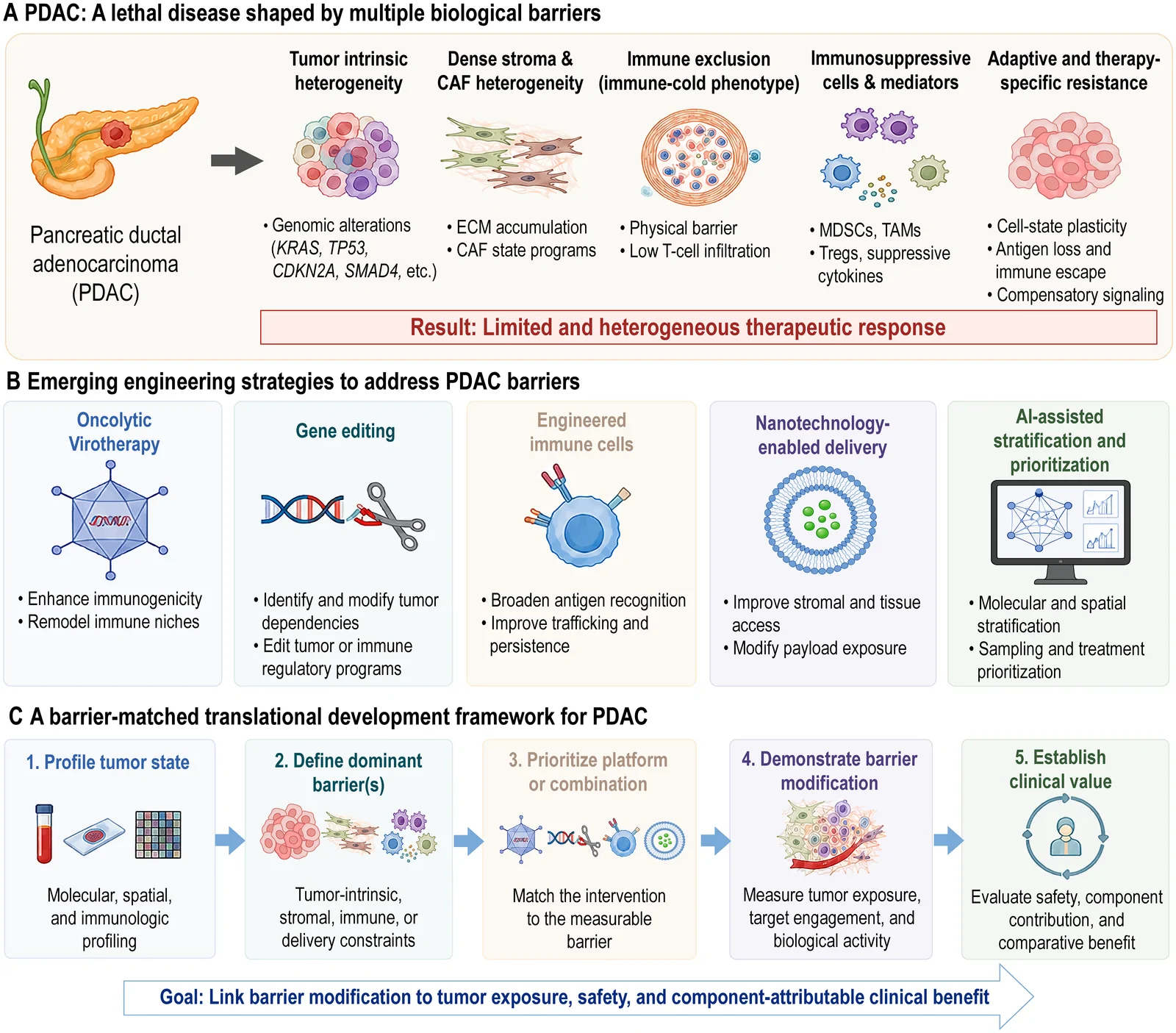
Barrier-matched translational development framework for pancreatic ductal adenocarcinoma. **(A)** Pancreatic ductal adenocarcinoma (PDAC) is shaped by tumor-intrinsic heterogeneity, dense stroma and context-dependent cancer-associated fibroblast (CAF) programs, immune exclusion, immunosuppressive cells and mediators, and adaptive or therapy-specific resistance, producing limited and heterogeneous treatment responses. **(B)** Oncolytic virotherapy, gene editing, engineered immune cells, nanotechnology-enabled delivery, and artificial intelligence (AI)-assisted stratification address different components of this barrier landscape. **(C)** The proposed framework links tumor profiling and dominant-barrier definition to platform prioritization, demonstration of tumor exposure and biological activity, and evaluation of safety, component contribution, and comparative clinical benefit. The framework is a translational-development tool rather than a validated patient-level treatment-assignment algorithm.

## 2 Complex biological landscape of PDAC

### 2.1 Epidemiological, clinical, and molecular characteristics

Pancreatic ductal adenocarcinoma (PDAC) accounts for more than 90% of pancreatic malignancies and remains one of the most lethal solid tumors (1). According to GLOBOCAN 2022, pancreatic cancer accounted for approximately 511,000 new cases and 467,000 deaths worldwide (3). The global burden is expected to rise further, driven largely by population ageing and growth. Early-stage PDAC is frequently asymptomatic, and effective population-wide screening is currently unavailable. Consequently, only approximately 10–20% of patients present with resectable disease, whereas 30–40% have locally advanced tumors and approximately half have metastatic disease (4). The overall 5-year relative survival is currently approximately 13.7% and decreases to approximately 3.4% for metastatic disease (5).

Surgical resection followed by systemic therapy remains the only potentially curative strategy. Modified FOLFIRINOX is an established adjuvant treatment for medically fit patients following resection (2, 6). For advanced disease, FOLFIRINOX, gemcitabine plus nab-paclitaxel, and liposomal irinotecan plus fluorouracil, leucovorin, and oxaliplatin (NALIRIFOX) are major first-line systemic options (2, 7–9). However, their benefits remain limited by cumulative toxicity, early recurrence, declining performance status, and the development of therapeutic resistance (1, 2).

PDAC is driven by a characteristic but heterogeneous set of genomic alterations. Activating *KRAS* alterations occur in more than 90% of conventional PDACs, whereas *TP53*, *CDKN2A*, and *SMAD4* are also frequently inactivated. Oncogenic *KRAS* sustains proliferative, metabolic, and inflammatory signaling; loss of *CDKN2A* facilitates cell-cycle progression; *TP53* inactivation promotes genomic instability and stress tolerance; and *SMAD4* loss disrupts canonical TGF-β signaling. A smaller subgroup harbors clinically relevant alterations in DNA-damage repair genes, including *BRCA1*, *BRCA2*, and *PALB2*, providing opportunities for molecularly selected treatment (1, 10).

Transcriptomic studies have further identified distinct but partially overlapping PDAC phenotypes. Collisson et al. described classical, quasi-mesenchymal, and exocrine-like subtypes (11); Moffitt et al. identified classical and basal-like tumor subtypes together with normal and activated stromal states (12); and Bailey et al. proposed squamous, pancreatic progenitor, immunogenic, and aberrantly differentiated endocrine–exocrine subtypes (13). Despite differences in nomenclature, these classification systems largely converge on an epithelial axis ranging from a differentiated, GATA6-high classical state to a poorly differentiated, GATA6-low basal-like or squamous state (14). Differences in genomic alterations and transcriptional subtypes contribute to interpatient heterogeneity, whereas the coexistence of classical, basal-like, and intermediate states within individual tumors reflects substantial intratumoral heterogeneity (14, 15). These states are not fixed or mutually exclusive and may change during tumor evolution or treatment. Epithelial–mesenchymal plasticity, regulated in part by SNAIL, ZEB, and TWIST family transcription factors, contributes to loss of epithelial differentiation, invasion, metastatic dissemination, and resistance to cytotoxic stress (16).

Molecular states in PDAC are accompanied by distinct immune and stromal contexts. Spatial and single-cell studies have identified the coexistence of malignant-cell programs with heterogeneous fibroblast, myeloid, and lymphoid populations. In treated and chemoresistant samples, shifts in tumor-cell state have been observed alongside inflammatory fibroblast enrichment and altered immune-cell programs (17). Integrated single-cell analyses similarly suggest that epithelial subtype and stromal composition evolve in a coordinated manner rather than as independent features (18). The functions and interactions of these stromal and immune populations are examined in greater detail in Section 2.2.

Molecular subtype is clinically relevant, although its role in treatment selection remains unsettled. Classical tumors generally retain epithelial differentiation, whereas basal-like tumors more frequently display epithelial–mesenchymal features and aggressive clinical behavior. In a large real-world cohort, strongly basal tumors were associated with shorter overall survival among patients receiving either FOLFIRINOX or gemcitabine plus nab-paclitaxel (19). A subsequent real-world analysis using the PurIST classifier suggested that patients with classical tumors may derive greater benefit from first-line FOLFIRINOX than from gemcitabine plus nab-paclitaxel (20). The two retrospective analyses identify subtype as a clinically relevant stratifier, but they do not yet establish routine treatment assignment because assay standardization and representative sampling remain unresolved.

PDAC resistance is therefore best understood as an interaction between tumor-cell genotype and the surrounding stromal and immune context. Experimental models show that microenvironmental signals can alter malignant-cell state and drug sensitivity without requiring new genomic alterations (21). These findings support treatment strategies that consider both tumor-intrinsic vulnerabilities and the surrounding stromal and immune context.

### 2.2 Dynamic stromal and immune barriers

PDAC is shaped by a spatially organized and treatment-responsive microenvironment rather than a fixed stromal barrier. Fibroblast, myeloid, and lymphoid programs vary across locations and disease states, such that physical exclusion, defective antigen presentation, and immune-cell dysfunction should be evaluated as related but distinct processes (22, 23).

#### 2.2.1 Fibroblast states and selective stromal intervention

The commonly described myCAF, iCAF, and apCAF populations are better viewed as archetypal programs than as fixed, mutually exclusive lineages. Tumor-proximal myCAFs preferentially express contractile and matrix-associated programs, whereas more distant iCAFs are enriched for inflammatory mediators (24). Their balance is signal dependent: IL-1 induces LIF–JAK–STAT signaling and favors the iCAF program, while TGF-β antagonizes this pathway and promotes myofibroblastic differentiation (25). Cross-species single-cell analyses identified a major histocompatibility complex class II (MHC-II)/CD74-associated apCAF state whose lineage stability and capacity for productive antigen presentation require functional validation (26). Mesothelial cells are one experimentally defined source of apCAFs capable of inducing antigen-specific regulatory T-cell differentiation (27). Pancreatic stellate cells contribute to this compartment but do not account for all fibroblast lineages (28, 29). The resulting picture is one of location- and signal-dependent plasticity with functional overlap among fibroblast states.

Mechanistic studies further show that CAF activity cannot be reduced to a shared pathway. Inflammatory-cell-derived CXCL3 promotes CXCR2-dependent myofibroblast conversion (30), whereas malignant-cell-derived TGF-β induces an autocrine AREG–EGFR/ERBB2 circuit in a subset of myCAFs (31). Broad stromal depletion also differs from state-selective intervention. Depletion of αSMA-positive myofibroblasts or reduction of epithelial Shh-dependent stroma produced more aggressive disease in distinct genetically engineered models (32, 33). By contrast, selective depletion of LRRC15-positive myofibroblasts reduced tumor growth through a CD8-dependent mechanism in the tested preclinical models (34). These findings distinguish broad ablation, selective state targeting, and stromal normalization, nominating state-selective intervention for clinical testing. The clinically relevant question is which state or circuit dominates in a given tumor, rather than how much stroma is present.

#### 2.2.2 Myeloid heterogeneity, antigen presentation, and immune exclusion

Human multimodal profiling identifies patient-specific monocyte, macrophage, and granulocyte states that coexist with variable T-cell dysfunction (35). A simple M1/M2 scheme therefore obscures lineage, activation state, and spatial context. In *Kras*-driven models, tumor-derived granulocyte–macrophage colony-stimulating factor (GM-CSF) expands suppressive myeloid populations, whereas selective depletion of granulocytic myeloid-derived suppressor cells (MDSCs) unmasks adaptive immunity (36, 37). These studies provide a causal link between tumor-cell signaling, myeloid accumulation, and T-cell exclusion rather than treating myeloid abundance as a descriptive correlate.

Antigen presentation is impaired at a separate but connected level. PDAC reduces conventional dendritic-cell abundance and function, limiting tumor-antigen transport and cross-presentation and weakening CD8-positive T-cell priming; restoration of conventional dendritic cell (cDC) number or function restrained disease in spontaneous models (38). Human and murine PDAC also show tumor-driven disruption of IRF8-dependent conventional type 1 dendritic cell (cDC1) development, with expansion of immature granulocytes at the expense of the cDC lineage and reduced tumor-specific CD8-positive responses (39). Myeloid suppression in PDAC therefore reflects both accumulation of inhibitory populations and loss of immune-stimulatory antigen-presenting cells. This distinction supports interventions directed at the dominant myeloid defect rather than the compartment as a whole.

#### 2.2.3 Treg-linked compensatory networks

Regulatory T-cell biology further illustrates why cell-specific mechanisms cannot be interpreted outside their stromal context. Mesothelial-derived apCAFs induced antigen-specific regulatory T cell (Treg) differentiation in defined models (27), linking fibroblast state to adaptive immune regulation. Yet Treg depletion accelerated spontaneous *Kras*-driven pancreatic carcinogenesis and triggered compensatory fibroblast and myeloid remodeling, whereas syngeneic transplant tumors responded differently (40). The divergent results indicate that disease stage and stromal architecture shape the effect of Treg-directed intervention; compensatory fibroblast and myeloid remodeling is part of the mechanism rather than a secondary consideration. The coordinated malignant-cell, stromal, and immune states that shape treatment responsiveness are summarized in **Figure 2**.

**Figure 2:**
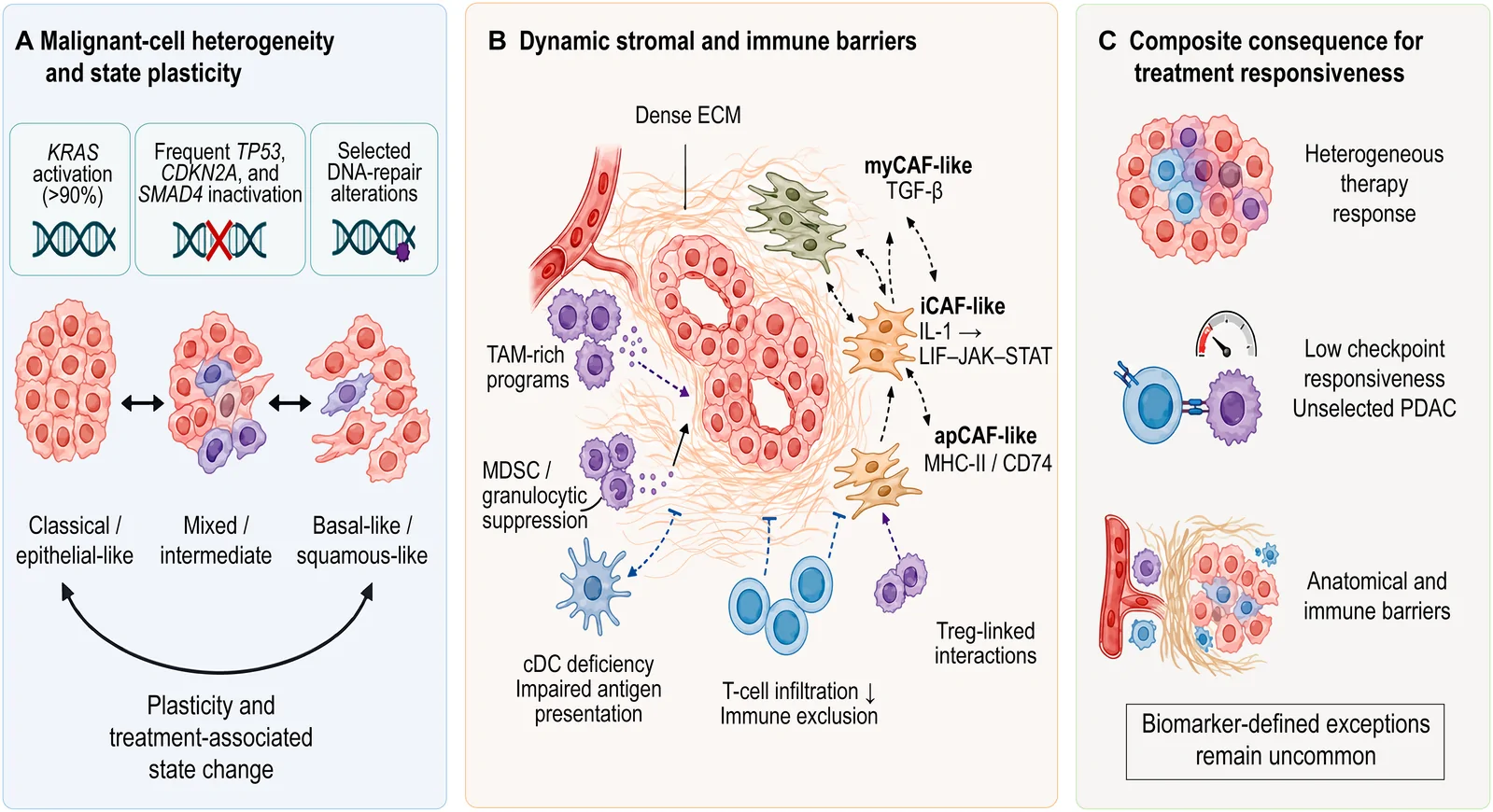
Dynamic molecular, stromal, and immune states shaping treatment responsiveness in pancreatic ductal adenocarcinoma. **(A)** Major genomic alterations and plasticity across classical/epithelial-like, mixed/intermediate, and basal-like/squamous-like malignant-cell states. **(B)** Dense extracellular matrix (ECM), context-dependent myofibroblastic CAF-like (myCAF-like), inflammatory CAF-like (iCAF-like), and antigen-presenting CAF-like (apCAF-like) programs, suppressive myeloid populations, conventional dendritic-cell deficiency, impaired antigen presentation, regulatory T-cell-linked interactions, and low T-cell infiltration form a spatially organized and treatment-responsive barrier network. **(C)** Their combined effects contribute to heterogeneous therapy response and low checkpoint responsiveness in unselected PDAC, while biomarker-defined exceptions remain uncommon.

### 2.3 Clinical immunotherapy landscape in PDAC

The low responsiveness of unselected PDAC to checkpoint blockade is consistent with the combined effects of T-cell exclusion, deficient antigen presentation, suppressive myeloid programs, and compensatory stromal signaling described above. Because these mechanisms vary among tumors and disease settings, clinical results require biomarker-specific interpretation.

#### 2.3.1 Checkpoint failure and biomarker-defined exceptions

In unselected PDAC, prospective trials establish a low-response baseline for checkpoint blockade. Single-agent ipilimumab produced no Response Evaluation Criteria in Solid Tumors (RECIST) responses among 27 patients (41). In a randomized phase II study, durvalumab alone and durvalumab plus tremelimumab yielded response rates of 0% and 3.1%, respectively, and the prespecified expansion threshold was not met (42). CheckMate 032 likewise found no responses with nivolumab alone or combined with ipilimumab; adding mitogen-activated protein kinase kinase (MEK) inhibition produced occasional responses, but the cohorts were small, nonconcurrent, and lacked a standard-treatment control (43). These trials define a low-response baseline in unselected PDAC rather than an absolute absence of benefit.

Microsatellite instability-high/mismatch repair-deficient (MSI-H/dMMR) is a rare, clinically actionable exception. PDAC series identified dMMR in 7/833 tumors and MSI-high and/or tumor mutational burden-high (TMB-high) in approximately 0.5% of cases (44, 45). The retrospective AGEO cohort of 31 evaluable patients with MSI/dMMR pancreatic adenocarcinoma reported an objective response rate (ORR) of 48.4%, supporting clinically meaningful activity in a biomarker-selected population (46). KEYNOTE-158 broadens the tumor-agnostic evidence base (47), but PDAC-specific evidence for TMB-high disease remains sparse.

#### 2.3.2 Vaccines and antigen-directed priming

Personalized RNA vaccination has produced high-magnitude neoantigen-specific T-cell priming, establishing immunogenicity in resected PDAC. In a postoperative phase I cohort, 8 of 16 evaluable patients developed high-magnitude neoantigen-specific T-cell responses (48). Long-term follow-up showed that some vaccine-induced clones persisted for approximately three years, extending the observation from initial priming to durable immune memory (49). Because vaccination was delivered within a multicomponent adjuvant regimen, recurrence and survival outcomes cannot be attributed to the vaccine alone.

*KRAS*-vaccine evidence is similarly dependent on trial design and disease setting. The 176-patient randomized, double-blind, placebo-controlled GI-4000 study showed no overall improvement in recurrence-free or overall survival (50). ELI-002 was instead evaluated in a single-arm, minimal residual disease cohort of 25 patients—20 with PDAC and five with colorectal cancer—and demonstrated *KRAS*-specific immune responses (51). The association between response magnitude and subsequent survival is hypothesis-generating. Read alongside the negative GI-4000 trial, these data make disease setting and clinical endpoint—not immunogenicity alone—central to interpretation.

#### 2.3.3 CD40 agonism, bispecific engagers, and dendritic-cell strategies

CD40 combinations have generated clinically measurable activity, but their added value over contemporary chemotherapy remains unresolved. PRINCE randomized 105 efficacy-evaluable patients among three experimental combinations but included no concurrent chemotherapy-only arm; comparison with historical one-year survival thresholds could not isolate the contribution of sotigalimab (52). Only the nivolumab/chemotherapy arm met its prespecified historical threshold, whereas neither sotigalimab-containing arm did. OPTIMIZE-1 reported a confirmed response rate of 40% (23/57) with mitazalimab plus mFOLFIRINOX, yet its single-arm design cannot establish superiority or added benefit over mFOLFIRINOX (53). The regimens therefore show clinical activity while leaving the contribution of CD40 agonism unquantified.

For soluble CD3 engagers, MEDI-565 established phase I feasibility in a mixed gastrointestinal cohort, although PDAC-specific activity was not estimable (54). Dendritic-cell studies likewise support feasibility and immunogenicity: adjuvant MesoPher induced immune responses in 38 selected patients (55), whereas REACtiVe-2 defined the limited response activity of the tested metastatic regimen (56). These clinical studies also show that no single modality addresses the full spectrum of PDAC resistance. The following sections therefore examine emerging platforms in relation to the biological or delivery constraints they are intended to modify and the evidence required to demonstrate that effect.

## 3 Oncolytic virotherapy

### 3.1 Biological rationale and engineering principles

Oncolytic viruses (OVs) are replication-competent agents designed or selected to preferentially infect malignant cells, produce direct lysis, and expose tumor antigens and danger signals that can recruit host immunity. Engineering may alter entry tropism, restrict replication, shield particles from antiviral factors, degrade extracellular matrix, or deliver immunomodulatory transgenes (57–59). These functions vary across viral backbones, and in PDAC their expression is shaped by receptor availability, tumor-cell antiviral programs, desmoplastic architecture, route of administration, and host permissivity. These variables explain why tumor-selective replication and immune amplification observed with one virus–host–route combination cannot be assumed for another. The major virus–host–route determinants and the evidence required for translation are summarized in **Figure 3**.

**Figure 3:**
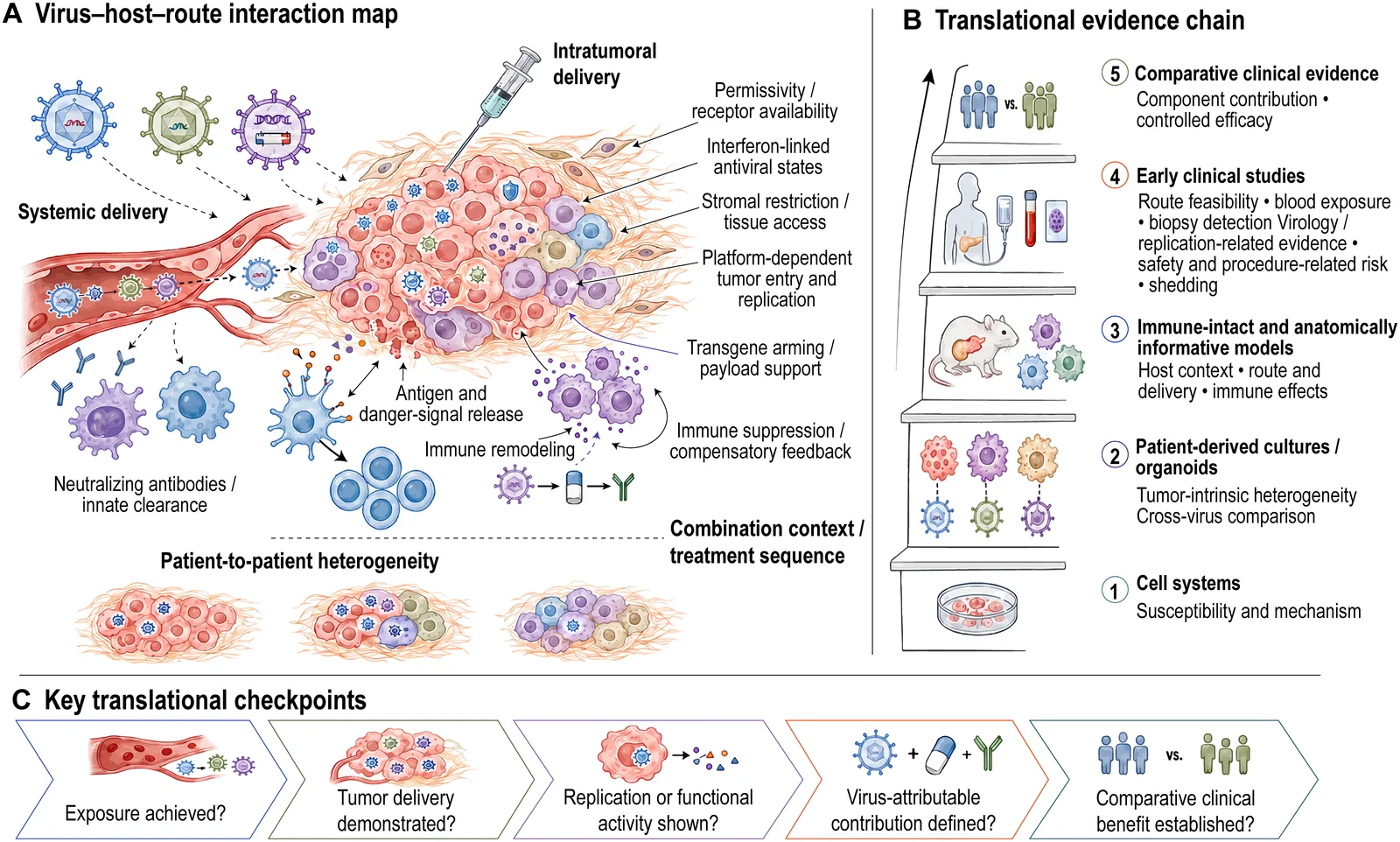
Virus–host–route determinants and translational evidence chain for oncolytic virotherapy in pancreatic ductal adenocarcinoma. **(A)** Oncolytic-virus performance depends on systemic or intratumoral delivery, tumor-cell permissivity, antiviral state, stromal access, neutralizing antibodies and innate clearance, platform-dependent entry and replication, transgene activity, immune remodeling, suppressive feedback, treatment sequence, and interpatient heterogeneity. **(B)** Evidence progresses from cell systems and patient-derived cultures or organoids to immune-intact and anatomically informative models, early clinical studies, and controlled comparative evidence. **(C)** Key translational checkpoints are systemic exposure, tumor delivery, replication or functional activity, virus-attributable component contribution, and comparative clinical benefit.

### 3.2 Preclinical evidence across translational barriers

#### 3.2.1 Tumor selectivity, systemic delivery, and intratumoral distribution

Retargeting can improve infection in selected human PDAC systems without establishing universal tumor specificity (**Table 1**). An intravenously administered mesothelin-targeted adenovirus produced persistent tumor genomes and lower liver genomes in mesothelin-high AsPC-1 xenografts and two subcutaneous PDAC patient-derived xenografts (PDXs) (60). The human xenografts support mesothelin-dependent tropism, whereas the permissive murine system shows that replication can be examined under intact immunity (60, 61). This complementary evidence strengthens the retargeting rationale but does not establish repeat systemic performance in a single immunocompetent, replication-permissive model.

**Table 1:** Representative adenovirus engineering, delivery, and translational evidence in pancreatic ductal adenocarcinoma.

Platform / engineering	Model / immune context / tumor site	Route	Main finding	Safety / PK	Evidence boundary	Ref.
Mesothelin-targeted Ad; fiber retargeting	AsPC-1 xenograft and two PDAC PDXs; immunodeficient, subcutaneous	IV	Persistent tumor genomes with lower liver genomes in selected mesothelin-high models.	Tumor/organ qPCR and human-serum neutralization; no complete PK.	Human tropism without intact antiviral immunity; repeat dosing unresolved.	(60)
Polymer-coated oAd-loaded MSC; cell carrier plus polymer shielding	AsPC-1 xenograft; nude mouse, subcutaneous	Repeated IV	Greater tumor localization and control than free virus or incomplete carrier controls.	24-h organ qPCR showed pulmonary trapping; no serial blood PK or formal toxicology.	Complex live-cell/virus manufacture; no intact antiviral immunity.	(62)
PBAE-coated AdNuPARmE1A; polymer shielding	Preimmune/passively immunized xenograft; partial anti-Ad immunity, subcutaneous	IV	Preserved infectivity under selected NAb pressure and reduced hepatic accumulation and innate responses.	NAbs, organ genomes, cytokines, liver enzymes, and histology; no concentration-time PK.	Partial antibody-pressure model is not repeat dosing in a permissive immunocompetent host.	(63)
IFN-expressing oAd plus chemoradiation; cytokine-armed combination	Syngeneic replication-permissive Syrian hamster; immunocompetent, subcutaneous	IT virus plus systemic therapy	Selected combinations improved tumor control and survival versus component arms.	Tumor viral measures, body weight, and serum chemistry; no serial systemic PK.	Ectopic tumors, species-specific translation, and a single local virus dose.	(66, 67)
Cytokine-armed oAd plus mesothelin CAR-T; virus-assisted cellular therapy	NSG human xenograft plus syngeneic surrogate; mixed immune context, subcutaneous	IT virus plus IV CAR-T	Improved CAR-T accumulation, function, and regression relative to component controls.	Preclinical combination assessment; no predictive CRS or off-tumor safety evaluation.	Two-product regimen; human CAR-T toxicity and manufacturing burden not reproduced.	(72)
KPC-I human-Ad permissivity model; murine tumor permissive to human Ad5	KPC-derived subcutaneous transplant; nude IV arm and C57BL/6 IT arm	IV or IT	Supported Ad5 infection, replication, and tumor control in model-specific arms.	Model-specific virology; formal toxicity and whole-body BD not reported.	Not a spontaneous GEMM; systemic delivery and intact immunity were not combined.	(61)

Endpoint viral genomes or organ qPCR are treated as biodistribution snapshots unless serial systemic exposure was measured. KPC-I denotes a KPC-derived transplanted model, not a spontaneous GEMM. “Not reported” means that the measurement was not described and does not imply absence. Ad, adenovirus; Ad5, adenovirus serotype 5; BD, biodistribution; CAR-T, chimeric antigen receptor T cell; CRS, cytokine-release syndrome; GEMM, genetically engineered mouse model; IFN, interferon; IT, intratumoral; IV, intravenous; MSC, mesenchymal stromal cell; NAb, neutralizing antibody; NSG, NOD-scid IL2Rγnull; oAd, oncolytic adenovirus; PBAE, poly(beta-amino ester); PDAC, pancreatic ductal adenocarcinoma; PDX, patient-derived xenograft; PK, pharmacokinetics; qPCR, quantitative polymerase chain reaction.

Delivery-focused studies demonstrate several platform-specific routes to systemic exposure. Repeated intravenous administration of polymer-coated, adenovirus-loaded mesenchymal stromal cells increased tumor localization and control relative to incomplete carrier controls in nude mice, but 24-hour organ quantitative polymerase chain reaction (qPCR) also documented pulmonary trapping and the approach adds live-cell manufacturing complexity (62). A poly(beta-amino ester) coating preserved adenoviral infectivity under selected neutralizing-antibody pressure and reduced hepatic accumulation and innate responses in passively immunized or preimmune xenograft models (63); this captures antibody pressure rather than the full immune context of repeat dosing in a replication-permissive host. Engineered vaccinia virus achieved broader spread after intravenous administration across subcutaneous, orthotopic, and disseminated pancreatic models (64), although the result is backbone-specific. Endpoint viral copies provide useful distribution snapshots but not concentration–time profiles. A phase I intravenous VCN-01 study later documented single-dose blood exposure, biopsy detection, neutralizing-antibody induction, and short-term shedding (65), demonstrating human tumor arrival and replication after one dose.

#### 3.2.2 Immune arming and combination strategies

Immunocompetent and replication-permissive hosts illuminate complementary components of OV activity, including viral replication, stromal context, antiviral immunity, and treatment sequencing (**Table 2**). Syrian hamsters permit human adenovirus replication and allowed intratumoral interferon-expressing adenovirus to be tested with chemoradiation in syngeneic subcutaneous tumors (66, 67). Syngeneic mouse studies offer broader immune reagents: engineered herpes simplex virus (HSV)-permissive and other syngeneic murine PDAC transplants revealed local changes in T-cell and myeloid compartments (68, 69), while KPC3 transplants showed that reovirus effects depended on route, pre-existing antiviral immunity, and treatment order (70, 71). Their strongest contribution is mechanistic comparison of route, antiviral memory, and treatment sequence. Vascular delivery through orthotopic human-like stroma remains a complementary validation task rather than a universal criterion for every model.

**Table 2:** Representative non-adenoviral mechanisms, immune modulation, and resistance evidence in pancreatic ductal adenocarcinoma.

Platform / biological question	Model / context	Route	Key finding	Translational boundary	Ref.
Immune modulation and combination context					
oHSV – immune remodeling and CD40L arming	Panc02-HVEM or other syngeneic murine PDAC transplants; immunocompetent, subcutaneous	IT	Local treatment changed immune composition; CD40L arming improved CTL-favorable control in selected models.	Engineered/model-specific permissivity and local dosing; no orthotopic systemic validation.	(68, 69)
Reovirus – preconditioning, sequence, route, and antiviral memory	KPC3 syngeneic immunocompetent transplants; subcutaneous or bilateral	IT/IV, experiment dependent	Reovirus alone did not control tumors; preconditioning enabled CD3-bispecific therapy, and antiviral memory could enhance efficacy.	Ectopic tumors; outcome depends on regimen, route, and host immunity.	(70, 71)
Vaccinia VVL-21 – systemic inter- and intratumoral spread	Subcutaneous, orthotopic, and disseminated pancreatic models; mixed immune context	Repeated IV	Improved spread and control versus parental virus across multiple anatomic models.	Backbone specific; endpoint copies or titers are not complete human PK.	(64)
MV-NIS plus anti-PD-1 – checkpoint combination and durability	Patient-derived cultures plus syngeneic preclinical PDAC model	IT MV-NIS plus systemic anti-PD-1	Immune remodeling and antitumor activity were observed but were transient.	Model-dependent measles permissivity; no systemic-delivery or durability validation.	(74)
Susceptibility, resistance, and patient heterogeneity					
VSV - resistance determinants and treatment-state dependence	Human or murine PDAC cell panels; in vitro	In vitro	Susceptibility varied by tumor state; adaptation improved replication, whereas chemoresistance or METTL3 depletion could restrict it.	Mechanistic cell studies; no in vivo delivery, PK, or safety validation.	(76–79)
Patient-derived culture screen - cross-virus response and biomarker discovery	Fourteen resected patient-derived PDAC cultures; five viruses; ex vivo	Ex vivo	No virus covered all samples; virus-specific transcriptional associations emerged.	No intact vascular, immune, stromal, PK, or prospective predictive context.	(80)
PDAC organoid screen - cross-patient and cross-virus heterogeneity	PDOs from 10 patients; four viral platforms; ex vivo 3D	Ex vivo	Different organoids responded to different viruses; most were sensitive to at least one.	No vasculature, intact immunity, systemic safety, or validated clinical prediction.	(81)
Host-compartment signaling and suppressive feedback					
STING - host immune activation versus tumor-cell permissivity	Syngeneic PDAC tumors and a defined tumor-cell STING/TBK1 model	IT or in vitro; model dependent	Host cGAMP enhanced DC/T-cell responses, whereas tumor-cell STING loss or TBK1 inhibition increased HSV replication in a separate setting.	The direction of effect depends on virus, cellular compartment, and intended intervention.	(73)
OV-induced suppressive feedback - IL-1β-positive monocyte/IL-6-positive CAF/DC axis	Syngeneic PDAC plus patient-derived ex vivo material	IT oHSV in syngeneic models	OV treatment induced a stromal/myeloid circuit that suppressed DC-mediated immunity; model-directed rescue was tested.	Mechanistic preclinical evidence; durability and human causal relevance remain unresolved.	(75)

Patient-derived cultures and PDOs capture selected tumor-intrinsic heterogeneity but do not establish systemic delivery, intact host immunity, whole-body safety, or clinical predictive validity. STING effects are compartment dependent: tumor-cell STING/TBK1 signaling may restrict replication in a defined setting, whereas host immune-cell STING activation may enhance antitumor immunity. CAF, cancer-associated fibroblast; CD40L, CD40 ligand; cGAMP, cyclic GMP–AMP; CTL, cytotoxic T lymphocyte; DC, dendritic cell; HSV, herpes simplex virus; IT, intratumoral; IV, intravenous; METTL3, methyltransferase-like 3; MV-NIS, sodium iodide symporter-expressing measles virus; OV, oncolytic virus; PD-1, programmed cell death protein 1; PDAC, pancreatic ductal adenocarcinoma; PDO, patient-derived organoid; PK, pharmacokinetics; STING, stimulator of interferon genes; TBK1, TANK-binding kinase 1; VSV, vesicular stomatitis virus.

Combination studies show that component activity and treatment order can change efficacy. In complementary NOD-scid IL2Rγnull (NSG) xenograft and syngeneic surrogate models, intratumoral cytokine-armed adenovirus increased the accumulation and function of intravenous mesothelin-directed CAR-T cells relative to component controls (72). Local reovirus alone replicated without controlling KPC3 tumors, whereas reovirus preconditioning followed by a CD3-bispecific antibody enabled regression; reovirus-specific T-cell memory could also augment treatment (70, 71). CD40L-armed HSV and local cGAMP plus HSV enhanced cytotoxic T-cell or dendritic-cell responses in selected syngeneic PDAC transplants (69, 73). Across these models, treatment sequence, antiviral memory, and compensatory stromal signaling altered the outcome (69–75). OV-induced remodeling can be immunostimulatory or suppressive, so immune recruitment alone does not establish the added value of a combination.

#### 3.2.3 Heterogeneity and resistance to virotherapy

Viral susceptibility in PDAC is heterogeneous and can shift with treatment state. Experimental evolution improved vesicular stomatitis virus (VSV) attachment and replication in resistant human PDAC cells while retaining *in vitro* selectivity against tested nonmalignant cells (76). Acquired gemcitabine resistance increased VSV resistance in some, but not all, matched cell pairs (77); separate murine PDAC panels showed substantial intertumoral variation (78), and *METTL3* depletion established an intrinsic antiviral state that restricted VSV replication in human PDAC cells (79). Prior treatment and cell state therefore emerge as virus-specific determinants of permissivity, while the adapted variants remain untested *in vivo*. Patient-derived systems extend this comparison across tumors. A screen of 14 patient-derived PDAC cultures against five viral platforms found that 12 cultures responded to at least one virus, yet no virus covered all samples; interferon-stimulated and cyclic GMP–AMP synthase (cGAS)-related associations also varied by platform and context (80). Complementary organoid studies captured additional tumor-intrinsic heterogeneity and variable viral susceptibility (81). These systems reveal patient- and platform-specific response patterns but do not reproduce vascular, immune, or systemic behavior. Stimulator of interferon genes (STING) further illustrates the importance of cellular context: tumor-cell STING loss or TANK-binding kinase 1 (TBK1) inhibition increased HSV replication in one PDAC model (82), whereas host-directed cGAMP enhanced dendritic-cell and T-cell responses with HSV in syngeneic tumors (73). Any STING-directed biomarker must therefore specify the virus, cellular compartment, and intended intervention.

### 3.3 Clinical evidence

Randomized evidence provides the clearest anchor for interpreting uncontrolled studies (**Table 3**). In the peer-reviewed randomized phase II trial of 73 patients with untreated metastatic pancreatic adenocarcinoma, adding intravenous pelareorep to carboplatin–paclitaxel did not improve the primary endpoint: median progression-free survival (PFS) was 4.9 versus 5.2 months (p = 0.6), and median overall survival was 7.3 versus 8.8 months (p = 0.68) (83). The trial separates biological immune activation from comparative clinical benefit in this regimen. VIRAGE offers a more recent randomized comparison of two intravenous VCN-01 doses added to gemcitabine plus nab-paclitaxel. Among 96 patients in the conference-reported full-analysis set, median PFS was 7.0 versus 4.6 months and median overall survival was 10.8 versus 8.6 months (84). The study remains an open-label phase IIb conference/registry update; the overall-survival log-rank p value was 0.055, and full peer-reviewed populations, multiplicity handling, adverse-event tables, and virology are unavailable. VIRAGE is therefore encouraging but incomplete comparative evidence, not confirmed survival benefit. Completed early studies mainly establish route feasibility, short-term safety, and biological or virological measurements. In the multicenter phase I VCN-01 study, 42 patients comprised the safety population and 26 had PDAC in chemotherapy-combination cohorts; one intravenous dose generated dose-related viral-genome exposure in blood, viral genomes in five of six sampled tumors, E1A protein in one PDAC metastasis, rising neutralizing antibodies, and mostly short-lived genome shedding (65).

**Table 3:** Published and updated clinical evidence for oncolytic virotherapy in pancreatic ductal adenocarcinoma.

Agent	Design / population	Route / comparator	Main result	Safety / virology	Evidence interpretation	Ref.
Ad5 double-suicide gene therapy plus chemotherapy	Single-center phase I, 3+3; 11 enrolled, 9 completed/evaluable	EUS-IT; no control	No DLT; 12-week response observations were exploratory.	Serum Ad DNA in four patients at week 8; detailed PK, shedding, NAb, and product-quality data not reported.	Local feasibility and safety only; no virus-attributable efficacy.	(88)
LOAd703 plus chemotherapy	Nonrandomized phase I/II, arm 1; 22 enrolled, 21 treated, 18 activity evaluable	IT; no control	ORR 44% and DCR 94% in the selected activity-evaluable cohort.	One transient grade 3 ALT DLT; repeated local dosing; anti-Ad antibodies and T cells increased; shedding not reported.	Activity cannot be attributed to LOAd703.	(85)
VCN-01 plus chemotherapy, local cohort	Phase I proof-of-concept; n = 8	Repeated EUS-IT; no control	Replication-associated and stromal/HA pharmacodynamic changes; efficacy not estimable.	Blood/biopsy viral findings; complete shedding and NAb profile not reported.	Local feasibility and biological activity.	(86)
Pelareorep plus carboplatin/paclitaxel	Peer-reviewed randomized phase II; 73 patients (36 vs 37)	IV; chemotherapy control	PFS 4.9 vs 5.2 months (p = 0.6); OS 7.3 vs 8.8 months (p = 0.68).	Immune activation without comparative benefit; detailed viral kinetics and shedding not reported.	Published randomized anchor; null efficacy result.	(83)
Pelareorep plus pembrolizumab and chemotherapy	Open-label phase Ib; 11 enrolled, 10 response evaluable	IV; no control	One PR and two SD in a small multi-agent cohort.	Mostly grade 1–2 flu-like events; optional-biopsy pharmacodynamics; no full PK or shedding.	Feasibility and biological activity only; component contribution unresolved.	(89)
T-VEC	Phase I dose escalation; 10 enrolled, 9 treated/analyzed	Repeated EUS-IT; no control	No objective responses; 4/9 SD; median OS 7.8 months.	Two grade 3–4 intra-abdominal infections requiring hospitalization; PK/shedding not reported.	Local feasibility with material procedure-related risk.	(87)
H-1PV (ParvOryx)	Open-label dose-escalation phase II; n = 7	IV ×4 then intralesional; later gemcitabine; no control	Safety, immune, and viral findings; response attribution not possible.	Tumor nucleic acids detected in selected patients; no active virus in saliva or urine; limited fecal excretion.	Small mixed-route feasibility and virology evidence.	(90)
VCN-01 plus or minus chemotherapy, systemic	Multicenter open-label phase I; 42 safety, 26 PDAC in combination cohorts	Single-dose IV; no randomized control	Exposure, biopsy/replication, NAb, shedding, and safety endpoints; responses confounded by chemotherapy.	Fever/flu-like illness common; one grade 4 AST/lipase event at highest dose; high baseline NAbs excluded; short-term shedding.	Human systemic PK/virology feasibility, not repeat-dose efficacy.	(65)
HF10 plus erlotinib/gemcitabine	Open-label phase I; 12 enrolled, 9 completed/evaluable	Repeated EUS-IT; no control	Response, PFS, and OS observations in a multimodal regimen.	No HF10-related DLT judged; PK, shedding, and NAbs not reported.	Feasibility only; activity not attributable to HF10.	(91)
VCN-01 VIRAGE	Randomized open-label phase IIb conference/registry update; 112 registered, 96 FAS	Two IV doses plus chemotherapy; chemotherapy control	PFS 7.0 vs 4.6 months; OS 10.8 vs 8.6 months; OS log-rank p = 0.055.	Manageable safety reported; complete peer-reviewed AE, PK, shedding, and analysis details pending.	Incomplete comparative evidence; survival benefit unconfirmed.	(84)
Pelareorep plus gemcitabine	Single-arm open-label phase II; 34 enrolled, 33 evaluable, 29 CBR evaluable	IV; no control	CBR 58% among 29; median PFS 3.4 months and OS 10.2 months.	Flu-like and hematologic toxicity; population PK, NAbs, and shedding not reported.	Historical single-arm activity; randomized evidence is the counterweight.	(92)

Single-arm ORR, DCR, PR, SD, PFS, or OS observations were not attributed to the virus when active co-therapy was administered. VIRAGE is a randomized phase IIb conference/registry update rather than a complete peer-reviewed trial report. Registered studies without mature PDAC-specific results and broad solid-tumor studies without separable PDAC results were excluded from the efficacy evidence pool. Ad, adenovirus; AE, adverse event; ALT, alanine aminotransferase; CBR, clinical benefit rate; DCR, disease control rate; DLT, dose-limiting toxicity; EUS, endoscopic ultrasound; FAS, full-analysis set; H-1PV, H-1 protoparvovirus; HA, hyaluronic acid; IT, intratumoral; IV, intravenous; NAb, neutralizing antibody; ORR, objective response rate; OS, overall survival; PDAC, pancreatic ductal adenocarcinoma; PFS, progression-free survival; PK, pharmacokinetics; PR, partial response; SD, stable disease; T-VEC, talimogene laherparepvec.

Patients with high baseline neutralizing antibodies were excluded, and repeat-dose exposure was not evaluated. The intratumoral LOAd703 study enrolled 22 patients; 21 received treatment and 18 were included in the activity-evaluable population (85). Repeated endoscopic ultrasound (EUS)-guided intratumoral VCN-01 in eight patients documented replication-associated and stromal pharmacodynamic changes (86). T-VEC provided local-delivery experience in nine treated patients, with no objective responses and two grade 3–4 intra-abdominal infections requiring hospitalization (87). Smaller Ad5 suicide-gene, pelareorep combination, HF10, and H-1 protoparvovirus (H-1PV) cohorts further define route, safety, virology, and evaluable denominators (88–92). Registered studies without mature PDAC-specific results do not yet contribute to the efficacy evidence base.

### 3.4 Translational constraints and priorities

Clinical translation depends on sustained tumor exposure within a tolerable safety window. For systemic programs, blood exposure, tissue distribution of viral genomes and infectious particles, biopsy evidence of replication, neutralizing antibodies, cellular antiviral immunity, and environmental shedding need to be interpreted together. Repeat-dose intravenous data remain absent for most systemic platforms (65), whereas local injection provides direct tumor access at the cost of lesion selection and procedural risk (87). Orthotopic or spontaneous models that combine intact immunity, viral permissivity, repeat systemic dosing, and matched component controls provide the most informative bridge to clinical design.

Manufacturing demands differ by product. H-1PV studies illustrate how stability, handling, environmental safety, and upstream scale-up can be incorporated into development (93, 94). Multitransgene constructs additionally require stable inserts, relevant potency assays, sterility, storage, and lot consistency. These attributes matter only insofar as they preserve exposure and biological activity at a tolerable dose.

## 4 Gene editing and engineered cellular immunotherapies

### 4.1 Gene editing in PDAC

#### 4.1.1 Functional genomic screens and target discovery

Clustered regularly interspaced short palindromic repeat (CRISPR)-based screening has sharpened target prioritization in PDAC; its principal contribution at this stage is discovery rather than direct therapeutic editing. An *in vivo* CRISPR screen conducted in a native pancreatic genetically engineered mouse model (GEMM) context identified *USP15* and *SCAF1* as modifiers of tumor progression, providing organ- and immune-context information unavailable from conventional cell-line screens (95). By contrast, genome-wide screening identified *PKMYT1* dependency, whereas subsequent intervention relied mainly on a small-molecule *PKMYT1* inhibitor tested in cell-derived and patient-derived xenografts (96). The two approaches are complementary: the GEMM preserves pancreatic and immune context, whereas the xenografts connect the candidate to human tumor biology but not an intact immune system. This strengthens target prioritization across biological contexts while keeping target discovery distinct from therapeutic CRISPR delivery.

#### 4.1.2 Therapeutic RNA targeting, genome editing, and delivery

Current PDAC therapeutic editing is at a delivery-dependent proof-of-concept stage, with distinct requirements for RNA targeting and permanent DNA modification. Capsid-optimized adeno-associated virus serotype 8 (AAV8)-CasRx reduced *KRAS*^G12D^ RNA and tumor growth in an immunodeficient orthotopic AsPC-1 model and PDXs; PDX treatment was intratumoral (97). Because CasRx targets RNA, this represents silencing rather than permanent DNA editing. Safety assessment was limited to body weight, selected liver histology, and transcriptomic analyses; whole-body distribution, repeat dosing, and long-term toxicity were not established. Exosome-mediated CRISPR/Cas9 plasmid delivery instead targeted DNA in KPC689-derived syngeneic subcutaneous and orthotopic transplants in immunocompetent mice (98). These syngeneic transplants preserve intact immunity but do not reproduce the spontaneous disease context of a GEMM. The subcutaneous experiment combined intravenous and intratumoral administration, preventing attribution to either route alone, whereas the orthotopic experiment used intraperitoneal dosing. Detection of Cas9 mRNA and guide RNA in tumors alongside reduced growth supports tumor delivery and biological activity (98). Accordingly, the two systems face different translational questions: AAV8-CasRx must sustain tumor-selective RNA silencing beyond locally treated models, whereas exosome-mediated CRISPR/Cas9 must demonstrate quantifiable tumor editing without unacceptable normal-organ or genome-wide off-target effects (97–99).

### 4.2 Engineered immune-cell therapies

#### 4.2.1 Early clinical evidence for CLDN18.2 CAR-T

Clinical evidence for CLDN18.2-directed CAR-T has advanced from isolated cases to a pooled 24-patient phase I/Ib analysis, while remaining early-phase and uncontrolled (100, 101). A pooled analysis of two multicenter, open-label phase I/Ib trials included 24 previously treated patients with pancreatic cancer: five had received one prior line and 19 at least two (101). The ORR was 16.7%, disease control rate (DCR) 70.8%, median PFS 3.3 months, median overall survival (OS) 10.0 months, and median response duration 9.5 months; carbohydrate antigen 19-9 (CA19-9) decreased by at least 30% in 17 patients. Most grade 3 or higher events were preconditioning-related hematologic toxicities, whereas cytokine release syndrome (CRS) and gastrointestinal events were mainly grade 1–2. These findings establish preliminary activity and short-term feasibility in a CLDN18.2-selected cohort. Interpretation remains limited by the uncontrolled design and by toxicity attribution after lymphodepletion; uncommon severe events, antigen escape, and gastric on-target/off-tumor effects remain undefined.

#### 4.2.2 Engineering around antigen, trafficking, and stromal barriers

Engineered-cell strategies have been used to extend recognition beyond malignant cells, improve trafficking, and engage the stromal compartment. Mesothelin-directed CAR-T cells secreting an anti-FAP/anti-CD3 T-cell-engaging molecule (TEAM), termed mesoFAP CAR-TEAM cells, combined tumor-cell and stromal targeting across mixed-antigen, metastatic, and CAF-containing patient-derived xenograft models (102). CXCR4 modification improved homing and activity of CLDN18.2 CAR-T cells in an immunocompetent syngeneic PANC02-A2 model (103), identifying chemokine-guided trafficking as a modifiable barrier. Induced pluripotent stem cell (iPSC)-derived anti-prostate stem cell antigen (PSCA) CAR macrophages localized to Capan-1 xenografts despite low circulating levels (104), extending the strategy to a myeloid effector platform. CAR-natural killer (NK) studies add an alternative cellular backbone: CD70-directed cells showed PDAC/CAF killing *in vitro*, whereas the reported *in vivo* platform test used irradiated NK-92 cells in Raji lymphoma (105); nintedanib enhanced mesothelin CAR-NK activity in selected PDAC/CAF models (106). Across these studies, added engineering broadened recognition or improved homing in the tested models. Durable tumor residence and toxicity were not assessed under clinically representative conditions.

#### 4.2.3 TCR-T and personalized TCR discovery

TCR approaches extend targeting to intracellular-antigen-derived epitopes, while therapeutic engineering and receptor discovery answer different questions. LAMC2-specific TCR-engineered T cells mediated human leukocyte antigen (HLA)-dependent cytotoxicity and suppressed tumor growth in preclinical models, establishing a therapeutic proof-of-concept for an HLA-restricted population (107). A separate single-cell TCR/RNA-sequencing and autologous-organoid workflow supported candidate-receptor discovery and ex vivo validation (108), without therapeutic infusion. The studies support different stages of development: an HLA-restricted therapeutic strategy and a receptor-discovery workflow. HLA coverage, normal-tissue cross-reactivity, persistence, and the predictive value of organoid testing remain unresolved.

### 4.3 PDAC-specific translational constraints

Editing and engineered-cell products encounter different bottlenecks in PDAC. AAV8-CasRx and exosome-mediated CRISPR/Cas9 must produce measurable activity in orthotopic pancreatic tumors without clinically relevant vector or editing exposure in normal organs (97–99). Engineered lymphoid and myeloid cells face a different problem: trafficking through fibrotic stroma, persistence within an immunosuppressive compartment, and maintenance of function despite heterogeneous antigen expression (102–104, 107, 108). In the CT041 studies, most grade 3 or higher events were related to lymphodepletion, complicating attribution to the cell product, while CLDN18.2 targeting also requires continued attention to gastric on-target/off-tumor toxicity (100, 101). The relevant product attributes consequently differ: editing systems require specificity and tissue-distribution measurements, whereas cellular products require antigen-dependent potency, trafficking, and persistence (99, 109). The complementary evidence domains and platform-specific translational constraints are summarized in **Figure 4**.

**Figure 4:**
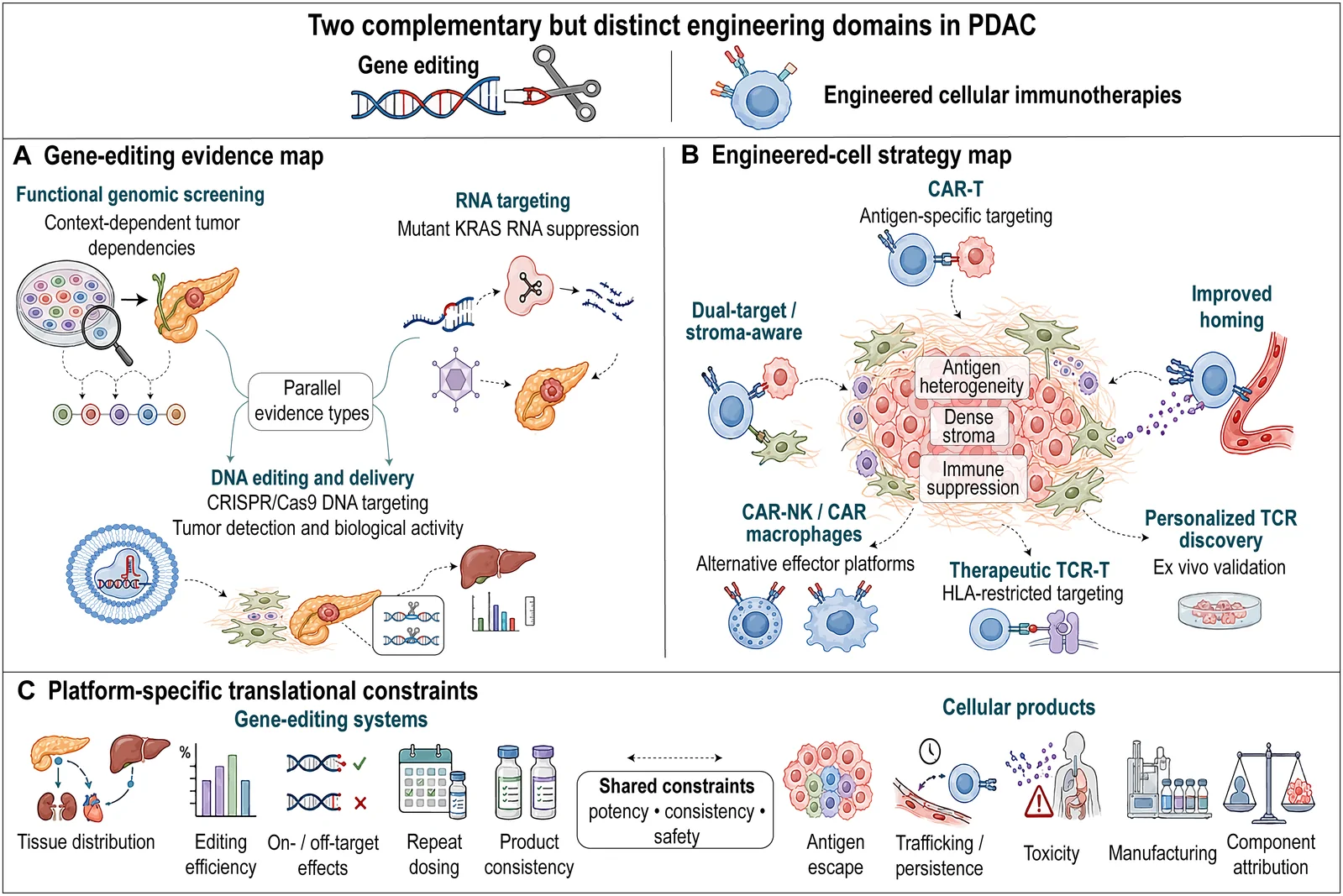
Complementary evidence domains for gene editing and engineered cellular immunotherapies in pancreatic ductal adenocarcinoma. **(A)** Gene-editing evidence includes context-dependent functional genomic screening, RNA-directed suppression, and DNA editing and delivery, which address distinct questions and should not be interpreted as a single linear development sequence. **(B)** Engineered-cell strategies include antigen-selected chimeric antigen receptor T cells (CAR-T cells), tumor- and stroma-aware designs, trafficking engineering, alternative CAR-engineered natural killer-cell or macrophage platforms, therapeutic T-cell receptor-engineered T cells (TCR-T cells), and personalized TCR discovery. **(C)** Translation requires platform-specific evaluation: tissue distribution, quantitative editing, off-target effects, repeat dosing, and potency for editing systems; and antigen heterogeneity, trafficking, persistence, toxicity, manufacturing, component attribution, and comparative benefit for cellular products.

## 5 Nanotechnology for TME-directed and immune-enabled delivery

The value of nanotechnology in PDAC lies in whether a formulation changes tumor access, payload exposure, or spatial distribution rather than in material complexity itself. Stromal penetration, immune-payload delivery, nucleic-acid transport, and viral shielding are distinct pharmacological tasks, and each requires a different readout. Fluorescence, pathway modulation, tumor regression, quantitative tissue distribution, and serial blood pharmacokinetics therefore cannot be treated as equivalent evidence; carrier kinetics may also differ from those of the active payload. In PDAC, where dense stroma and immune exclusion distort simple accumulation measures, a useful delivery gain must persist in anatomically and immunologically informative models without unacceptable normal-organ exposure (**Figure 5**).

**Figure 5:**
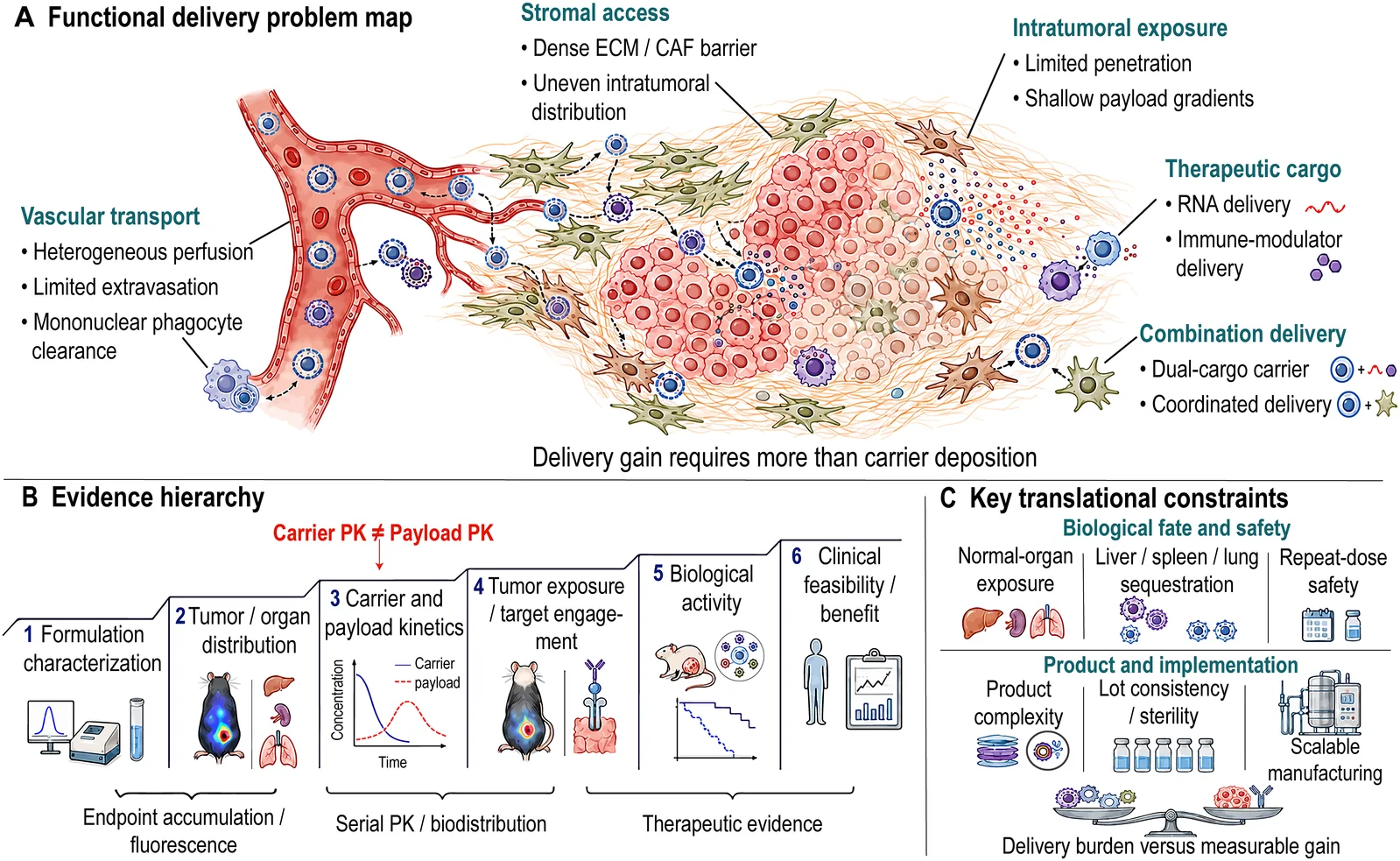
Functional delivery problems and evidence hierarchy for nanotechnology in pancreatic ductal adenocarcinoma. **(A)** Nanotechnology may address heterogeneous vascular perfusion, limited extravasation, mononuclear phagocyte clearance, stromal and tissue access, intratumoral payload exposure, immune or nucleic-acid cargo delivery, and coordinated combination delivery. **(B)** Evidence should progress from formulation characterization and endpoint distribution snapshots to separate carrier and payload pharmacokinetics, tumor exposure and target engagement, biological activity in relevant models, and clinical feasibility or measurable benefit. **(C)** Translational evaluation must account for normal-organ exposure, liver, spleen, and lung sequestration, repeat-dose safety, product complexity, sterility, lot consistency, scalable manufacture, and whether delivery gain justifies implementation burden.

### 5.1 Improving penetration through the desmoplastic stroma

Evidence for improved stromal access is strongest when a defined transport mechanism is paired with tumor-versus-organ measurements. Bacteria-based designs used either active Escherichia coli Nissle 1917 (EcN) motility with collagenase release (110) or bacteria-responsive nanoparticle disassembly (111) to increase access in orthotopic immunocompetent and subcutaneous Panc02 syngeneic models. These experiments demonstrate transport gains in specific models while also raising questions about bacterial persistence, clearance, and product control. The gold-nanoparticle study quantified tissue gold after a single intravenous dose (112), showing measurable tumor exposure together with substantial reticuloendothelial-organ uptake. Because distribution and efficacy were tested in different models, its principal value is quantitative exposure rather than gemcitabine pharmacokinetics (PK). Nanoparticles functionalized with fibronectin-binding peptide A5 and partially shielded by red blood cell vesicles extended the comparison to stromal reprogramming in syngeneic subcutaneous and orthotopic Panc02 tumors, where retinoic acid delivery increased intratumoral doxorubicin distribution (113). Active transport and stromal modification can therefore improve access, but the magnitude of the gain remains dependent on route, anatomy, and whether carrier or payload is measured.

### 5.2 Delivering immune-modulating payloads

Immune-enabled delivery is best evaluated through measured immune changes and dependence on combination therapy rather than a binary “cold-to-hot” label. Local ZDHHC9 small interfering RNA (siRNA) nanoparticles plus anti-PD-L1 generated CD8-dependent immune effects and tumor control in subcutaneous Panc02 or KPC syngeneic tumors (114). A pyroptosis/cyclooxygenase-2 (COX-2)/checkpoint platform produced broader myeloid and lymphoid changes and completely suppressed a second subcutaneous implant through day 30 in a Panc02 model (115). Systemic CB-5083/miR-142/R848 co-delivery linked endoplasmic-reticulum stress and Toll-like receptor 7/8 (TLR7/8) signaling to immune-cell changes and rechallenge responses in subcutaneous Panc02 tumors (116). The anti-CD40/anti-CLDN18.2 nanoengager added antigen-directed macrophage recognition in a CD40-humanized model, with higher tumor fluorescence alongside liver, spleen, and lung uptake (117). These studies show that nanodelivery can support local gene silencing, coordinated immune activation, and antigen-directed engagement. The evidence is strongest for mechanism in subcutaneous models, where the contribution of individual payloads and the behavior of the complete carrier remain difficult to separate.

### 5.3 Nucleic-acid delivery and clinical benchmarks

RNA delivery is most informative when carrier distribution is separated from intact-payload exposure and repeat-dose safety. The tLyp-1 siRNA-lipid nanoparticle (LNP) increased tumor accumulation relative to an untargeted control in subcutaneous CFPAC-1 xenografts, but hepatic predominance, spleen and lung exposure, and treatment-group deaths limit interpretation of tumor targeting without intact-siRNA pharmacokinetics (118). This study provides PDAC-specific evidence of target-dependent accumulation while placing normal-organ exposure and repeat dosing within the same pharmacological question.

Clinical benchmarks show that delivery systems can mature through different routes. NAPOLI 3 established randomized phase III benefit for a regimen containing liposomal irinotecan (9). Locally implanted siG12D-LODER defined procedural safety and tolerability in 59 patients, although its small randomized cohort did not show a significant overall-survival difference (119). Systemic iExo*Kras*^G12D^ provided early target-engagement and safety data without dose-limiting toxicity in phase I testing (120). These studies provide different benchmarks: randomized benefit for a liposomal chemotherapy regimen, procedural feasibility for a local implant, and early target engagement for systemic exosome delivery. None validates multifunctional immune nanoplatforms as a class. U.S. Food and Drug Administration (FDA) guidance places biological fate, tissue distribution, immunogenicity, and manufacturing control within the same development framework (121).

## 6 Artificial intelligence for molecular stratification and treatment prioritization in PDAC

### 6.1 Molecular and spatial stratification

Routine histology offers a practical starting point for converting PDAC molecular heterogeneity into reproducible trial strata. PACpAInt was trained in 202 resected tumors and tested in four independent cohorts comprising surgical specimens and liver biopsies; basal–classical discrimination yielded area under the receiver operating characteristic curves (AUCs) of 0.81–0.86 in surgical cohorts and 0.85 in biopsies (122). Across 598 patients and 1,796 slides, the model also exposed sampling-relevant heterogeneity: 30 of 77 RNA-classified classical tumors contained at least one slide predicted as basal-like, and these mixed cases had shorter survival than uniformly classical tumors. A bulk transcriptomic label may therefore conceal a spatially distinct component relevant to enrollment and tissue sampling. A separate public-data analysis extended stratification to model-defined immune states and candidate nomination; because neither the clusters nor the nominated drugs were tested against treatment response, its role is hypothesis generation (123). Pathology and multi-omic classification may therefore inform enrollment and sampling, while spatially discordant regions remain a source of classification uncertainty.

Spatial analysis provides a more direct bridge to immune intervention by resolving treatment-associated cell states within their tissue neighborhoods. In resected human PDAC specimens from treatment-naïve and neoadjuvant CD40-agonist cohorts, machine learning linked treatment exposure to interpretable T-cell changes across 306 regions from 29 patients, encompassing nearly 2.5 million cells and more than 1,000 regional microenvironmental features (124). Separate models for histopathologic regions used leave-one-patient-out validation, preventing regions from the same patient from being divided across training and test folds. Treatment-status AUCs were 0.87–0.90, while an immune-aggregate model classified longer versus shorter disease-free survival with an AUC of 0.77. Reduced canonical exhaustion and enrichment of CD44-positive CD4-positive Th1 states within proliferative, antigen-experienced, and cytotoxic immune neighborhoods provided an interpretable biological context for the classifier. Because the cohorts were small and no independent treatment dataset was available, the result is best interpreted as a spatial biomarker hypothesis rather than a response classifier

### 6.2 Treatment and combination prioritization

AI can support treatment choice at two complementary levels: reducing an experimental search space and identifying treatment-specific patient groups. In PANC-1 cells, three computational workflows used 496 measured combination matrices to rank approximately 1.59 million virtual drug pairs; 88 nominated combinations were tested in the same assay system, and 51 met the prespecified synergy criterion (125). This computation-to-experiment design enriched active pairs rather than merely scoring untested candidates. At the patient-cohort level, drug-specific transcriptomic signatures were retrospectively evaluated in 343 participants from PRODIGE-24; 164 were classified as matched to the treatment received, and matched mFOLFIRINOX or gemcitabine groups had longer disease-free survival than unmatched or predicted-resistant groups (126). The analysis links signatures to particular chemotherapy components rather than generic prognosis. At their current evidence levels, one study shows that AI can enrich experimental combination screening, whereas the other supports retrospective treatment matching; neither establishes prospective treatment assignment (125, 126).

### 6.3 Validation and clinical deployment

Across these studies, AI contributes at three linked stages: defining tissue states, narrowing experimental choices, and generating treatment-matching hypotheses (122–126). Its clinical value depends on external reproducibility and on whether model-guided decisions improve outcomes. Patient-level separation in multi-slide or multi-region datasets, cross-site calibration, prespecified assay procedures and thresholds, and a clear distinction between prognosis and treatment effect are the essential validation requirements. A comparative trial of model-guided versus predefined treatment assignment would provide the direct test of clinical utility.

## 7 Challenges and future perspectives

### 7.1 Matching therapeutic platforms to dominant PDAC barriers

Viewed through a barrier-matched framework, the performance of a modality depends on whether it addresses the dominant biological or delivery constraint in a given tumor, model, and treatment setting. PDAC resistance is not a single cold, fibrotic phenotype: tumor-cell subtype and plasticity, stromal organization, and immune-cell state vary across patients and can shift during treatment (19–21, 34, 40). The available platforms therefore serve different functions. Oncolytic viruses can combine local infection and antigen release with transgene expression (57, 58); engineered cells can broaden antigen recognition or improve trafficking and stromal engagement (102, 103); nanocarriers can modify tissue access and payload exposure (110, 118); and AI can define molecular or spatial states for stratification and sampling (122, 124). Their performance depends on antigen, route, host, and anatomical context. Barrier matching is a basis for prioritization, not a treatment-assignment rule. Platform selection begins with a measurable dominant barrier and evidence that the proposed intervention has modified it; manufacturing and delivery demands remain part of that decision.

### 7.2 Linking tumor exposure to clinical benefit

Selected programs already provide parts of a path from administration to clinical effect. Human OV studies have established route-specific blood exposure, biopsy detection, virological activity, and local feasibility (65, 87, 88, 90, 91). Editing and cellular programs contribute target-engagement, distribution, and early safety data (97, 101, 104), while nanocarrier studies add quantitative distribution and early clinical feasibility (112, 119, 120). Different models answer different questions: cell systems test susceptibility and mechanism, patient-derived cultures capture tumor-intrinsic heterogeneity, immunocompetent and orthotopic models address host and anatomical constraints, early clinical cohorts establish route and tolerability, and randomized trials estimate benefit. Evidence is strongest when administered dose can be linked to tumor exposure, active product or payload, target engagement, biological activity, and the added benefit of the investigational component. T-VEC and siG12D-LODER illustrate why route feasibility and delivery cannot substitute for safety or survival benefit (87, 119). Larger trials are difficult to justify while a critical step in this sequence remains unaddressed.

### 7.3 Biomarker selection and attribution in combination trials

Biomarker-defined enrollment and trials that separate the contribution of each component can convert heterogeneous early findings into testable treatment decisions. Molecular subtype and MSI/dMMR/TMB may inform clinical stratification (19, 20, 44–47). Minimal residual disease (MRD) and CLDN18.2 represent disease-setting and antigen-selection biomarkers (51, 100, 101), while patient-derived viral susceptibility and pathology- or spatially derived AI states may guide platform selection or tissue sampling (80, 81, 122, 124). Low checkpoint activity in unselected PDAC (41–43) and the negative GI-4000 trial (50) support selected enrollment and efficacy endpoints beyond immunogenicity, whereas early CLDN18.2 CAR-T data represent preliminary activity requiring controlled confirmation (100, 101). In multimodal regimens, each component needs a defined role and an interpretable contribution. The randomized pelareorep result provides a negative comparator (83), VIRAGE remains incomplete comparative evidence (84), and CD40 combinations lack a chemotherapy-only arm (52, 53). Because biomarker performance and component effects remain platform specific, their value must be tested within the intended regimen and population. The practical response is to prespecify biomarker strata and sampling rules, use contemporaneous comparators, and include matched component or sequence controls, including when AI prioritizes combinations or treatment assignment (125, 126).

### 7.4 Balancing engineering complexity with clinical use

Advanced engineering is valuable when it adds a clinically relevant function, such as local transgene expression, combined targeting, improved delivery, a safety switch, or data-supported treatment selection. The benefit must be weighed against the work required to reproduce it. Product identity, potency, stability, and comparability determine whether biological activity survives manufacture and repeat dosing, while normal-organ exposure and procedural demands shape the attainable safety window (93, 94, 99, 109, 121). For AI-supported decisions, assay standardization, performance across sites, and prespecified rules for model updating determine whether outputs remain stable enough for clinical use (122, 124, 126). These requirements differ across platforms but serve the same purpose: preserving an added function without losing interpretability. Feasibility in a specialized early-phase setting does not establish routine use, particularly when multiple products, local procedures, or adaptive models are involved. Added complexity is justified only by a consistent improvement in tumor exposure, potency, safety, clinical benefit, or practical delivery.

## 8 Conclusion

Pancreatic ductal adenocarcinoma remains difficult to treat because tumor-cell plasticity is coupled to stromal restriction, immune exclusion, defective antigen presentation, and limited drug or vector exposure. Emerging platforms should therefore be judged not by engineering complexity alone but by whether they modify a measurable barrier in a biologically and clinically relevant setting. Oncolytic viruses, gene-editing systems, engineered immune cells, nanocarriers, and AI-based stratification each address different parts of this problem, yet most evidence remains preclinical or early phase. Randomized and controlled data are limited, and early feasibility, immune activation, or tumor delivery cannot be assumed to confer added clinical benefit.

A barrier-matched translational framework offers a more useful basis for development. For each platform, administered dose should be linked to tumor exposure, target engagement, biological activity, safety, and the incremental contribution of the investigational component. Biomarker-defined enrollment, component-resolved trial design, and product-specific assessment of delivery, potency, manufacturing, and scalability are therefore central to further progress. This framework is not a validated treatment-assignment algorithm. Its value will depend on prospective evidence that modifying a defined PDAC barrier improves clinical outcomes with acceptable safety and implementation burdens.
